# Expression and purification of the p75 neurotrophin receptor transmembrane domain using a ketosteroid isomerase tag

**DOI:** 10.1186/1475-2859-11-45

**Published:** 2012-04-17

**Authors:** Qingxin Li, Angela Shuyi Chen, Shovanlal Gayen, CongBao Kang

**Affiliations:** 1 Institute of Chemical & Engineering Sciences, Agency for Science, Technology and Research, Singapore, 627833, Singapore; 2 Experimental Therapeutics Centre, Agency for Science, Technology and Research (A*STAR), 31 Biopolis Way #03-01, Nanos, Singapore, 138669, Singapore

**Keywords:** Membrane proteins, Ketosteroid isomerase, p75 neurotrophin receptor, Thrombin

## Abstract

**Background:**

Receptors with a single transmembrane (TM) domain are essential for the signal transduction across the cell membrane. NMR spectroscopy is a powerful tool to study structure of the single TM domain. The expression and purification of a TM domain in *Escherichia coli* (*E.coli*) is challenging due to its small molecular weight. Although ketosteroid isomerase (KSI) is a commonly used affinity tag for expression and purification of short peptides, KSI tag needs to be removed with the toxic reagent cyanogen bromide (CNBr).

**Result:**

The purification of the TM domain of p75 neurotrophin receptor using a KSI tag with the introduction of a thrombin cleavage site is described herein. The recombinant fusion protein was refolded into micelles and was cleaved with thrombin. Studies showed that purified protein could be used for structural study using NMR spectroscopy.

**Conclusions:**

These results provide another strategy for obtaining a single TM domain for structural studies without using toxic chemical digestion or acid to remove the fusion tag. The purified TM domain of p75 neurotrophin receptor will be useful for structural studies.

## Background

Membrane proteins play important roles in signal transduction across membranes of cells or organelles. Approximately 30% of the coded proteins from genomes are membrane proteins [1]. Additionally, an estimated over 50% of commercially available drugs target membrane proteins [2]. Structural studies of membrane proteins will provide information to understand their functional mechanisms and a structural basis for drug design. Currently structural information of membrane proteins still remains limited due to various difficulties. One of these difficulties is obtaining large quantity of protein necessary for structural studies using X-ray crystallography or NMR spectroscopy [3].

Compared with proteins with multiple-TM regions, many membrane proteins contain a single, alpha-helical TM domain that is important for signal transductions as seen in neurotrophin receptors [4]. The TM domain in this class of proteins has approximately 20–40 amino acids that are important for signal transduction through the interaction with other proteins or the formation of homo-oligomers. NMR spectroscopy is an important tool used to study the structures of these domains because their small size and the difficulties inherent in their crystallization [5,6]. To perform structural studies of proteins or peptides using NMR spectroscopy, isotopic labels (^15^ N and ^13^ C) are frequently incorporated because they increase the signal sensitivity. Additionally, the structures of some peptides and proteins can be determined using hetero- and homo-nuclear experiments when the peptides were folded into membrane-mimicking systems [7,8].

Chemically synthesizing a peptide corresponding to a TM domain of a protein is challenging due to the hydrophobic nature of the amino acids present in membrane proteins [9]. In addition, the peptide/micelle complex present as a protein with a high molecular weight, which makes isotopic label of the peptide necessary for structural determination using NMR spectroscopy. The expression and purification of a protein with isotopic labels in *E.coli* for NMR studies is economical and has been successfully used in many structural studies. Due to difficulties in the expression, detection and purification of small peptides, tags were used to increase yields or aid in the purification of recombinant protein from *E.coli* cells [10]. In membrane protein purification, there are two classes of tags that may be applied. One type of tags comprises proteins or parts of protein sequences such as the mistic and Bcl-xL tags that were shown to facilitate membrane protein expression and folding on the cell membrane [11,12]. The other type of tags comprises affinity tags such as poly-histidine tags, which are used to aid in the purification of target proteins. When proteins with multiple-TM regions were expressed in *E.coli*, the folding of the recombinant protein in the *E.coli* membrane was an important consideration because the folding may be affected by many factors [13]. However, for the structural study of a peptide derived from a single, helical TM domain of a membrane protein, refolding can be easily conducted because protein can be refolded during purification using a membrane-mimicking environment such as micelles [14].

**Figure 1:**
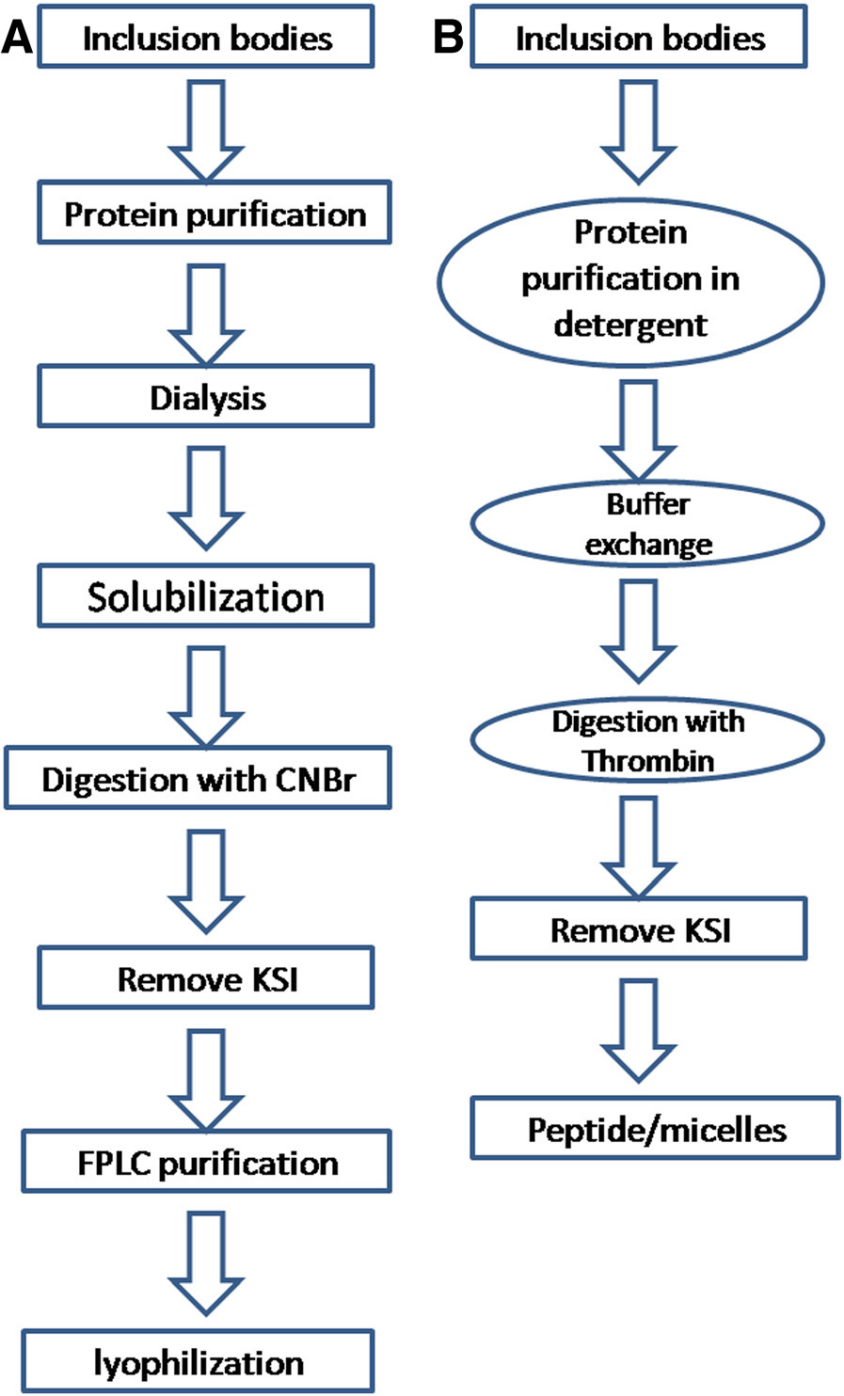
**The protein purification scheme using a KSI tag.** (**A**) Peptide purification using a CNBr cleavage. (**B**) Purification of a peptide from a TM domain using a KSI tag and thrombin cleavage.

Ketosteroid isomerase (KSI) was originally used in the expression of short peptides and is present in the commercially available pET31b vector. KSI was used to drive the target peptides into inclusion bodies so that the peptide yield and purity were increased. The procedure for using a KSI tag in a peptide purification normally includes recombinant protein over expression, inclusion body solubilization, protein purification, urea removal by dialysis, KSI tag removal using cyanogen bromide (CNBr) or acid, peptide purification using an FPLC system, solvent removal, peptide refolding in detergent when the peptide is a membrane protein or from a TM domain [15]. The normal procedure is summarized in Figure 1A. CNBr was used to break the peptide bond at the C-terminus of a methionine (Met) residue, which was then used to obtain a peptide fused with KSI because no Met residues are present in the KSI sequence. Because this procedure includes the application of toxic reagents, special concerns need to be taken into account to avoid toxic effects on the environment. In addition, Met residues are sometimes present in TM domains; thus, point mutations need to be made when CNBr is used to remove the KSI tag. A modified procedure to express short peptides corresponding to the TM domain of a membrane protein when KSI is incorporated will be helpful for structural studies. Herein, we describe a procedure for the expression of the TM domain of the p75 neurotrophin receptor (p75^NTR^) using a KSI tag (Figure 1B). To avoid the CNBr cleavage of the fusion protein, we added a thrombin cleavage site between KSI and the target protein. Thrombin is a protease shown to be active in almost all commercially available detergents and commonly used in structural studies [16]. We demonstrated that the KSI fusion protein can be folded on resin in a buffer containing micelles and that the fusion tag can be cleaved by the protease. Thus, the urea removal and CNBr cleavage steps can be omitted.

## Results

### Selection of the p75^NTR^ TM domain for expression

**Figure 2:**
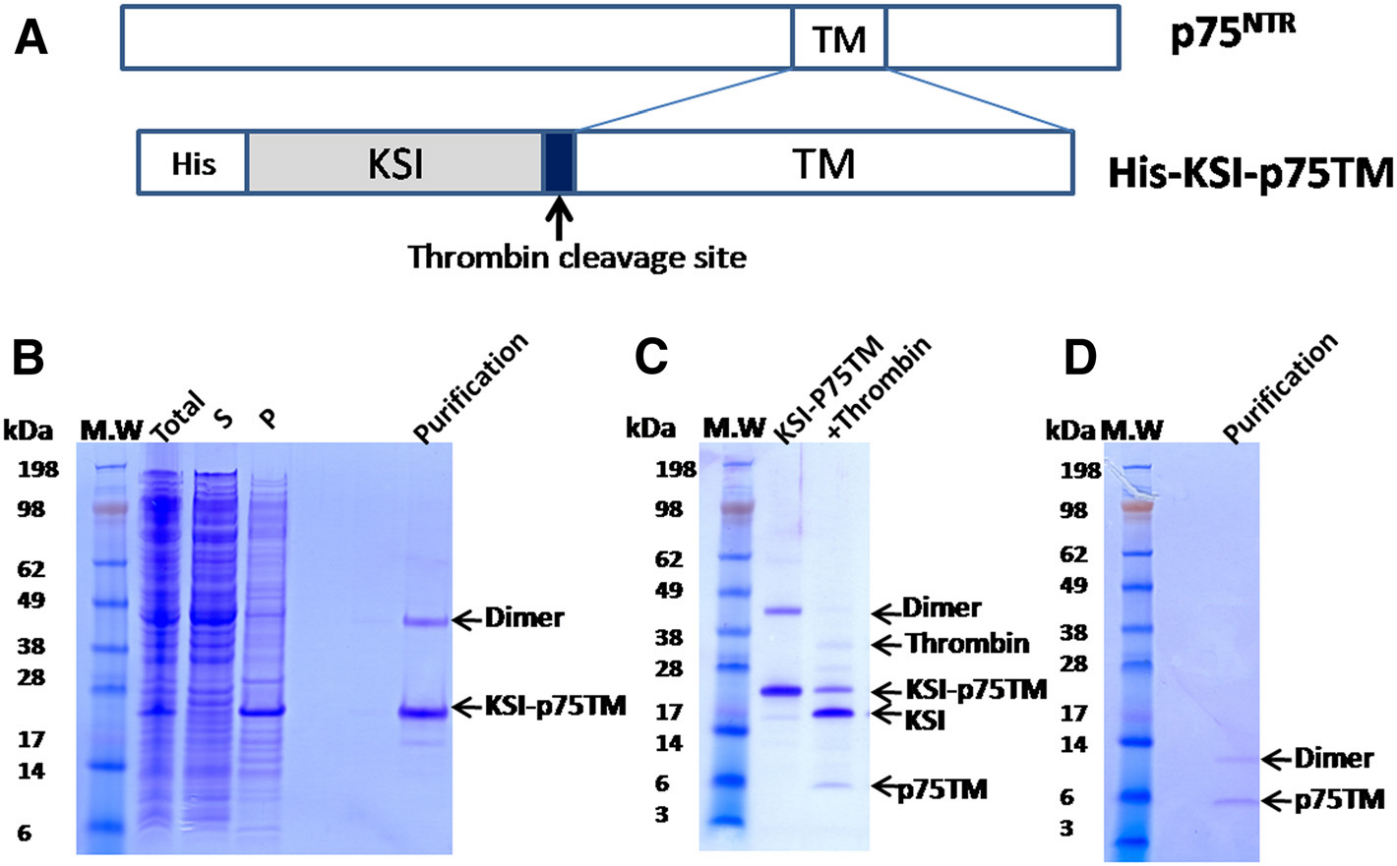
**Purification of the TM domain of p75**^**NTR**^**.** (**A**) The construct used for protein expression. (**B**) Expression of the protein fused with KSI. Total is the total cell lysate after induction, S is the cell lysate supernatant after centrifugation at 40,000 × g for 20 min at 4°C and P is the pellet after centrifugation and resuspension in the urea buffer. (**C**) Thrombin digestion of the purified protein. p75TM indicates the TM domain of p75^NTR^. (**D**) SDS-PAGE analysis of purified p75TM from gel filtration chromatography in a buffer containing 20 mM sodium phosphate, pH 6.5, 2 mM DTT and 0.1% DPC.

The p75^NTR^ protein is a membrane protein of the tumor necrosis factor receptor superfamily and it interacts with neurotrophins, which play important roles in the development and function of nervous system. p75^NTR^ is a type I TM receptor containing an intracellular domain, a TM domain and a type II consensus death domain that is a protein motif affecting apoptosis through protein-protein interactions [17]. Signal transduction through p75^NTR^ was proposed to include ligand binding and conformational changes in the TM domain [18,19]. The ligand-dependent recruitment in the extracellular region affects the TM domain, which affects the structure of the death domain, and therefore its ability to regulate the downstream signaling pathways. Previous studies showed that the TM domain is necessary for the dimerization of the receptor through formation of a disulfide bond between the Cys257 residues [19]. To understand the structural information of this domain, the cDNA encoding the KSI sequence and residues S239 to S287 containing the TM of p75 ^NTR^ were cloned into pET29 (Figure 2A) to ensure correct folding of the TM domain. During gene synthesis by GenScript, a thrombin cleavage site was introduced between the TM domain and KSI with a poly-histidine tag at the N-terminus (Figure 2A).

### Protein purification

We expressed the fusion protein as we did previously for other membrane domain proteins [20]. The protein was induced and expressed at 37°C overnight to facilitate its expression into inclusion bodies, which was confirmed by SDS-PAGE analysis (Figure 2B). Normally, KSI is purified into a buffer containing urea, however, in our study, we purified the fusion protein into a buffer containing detergent. The fusion protein was first dissolved into a urea buffer, then the urea was removed and the detergent exchange was performed. The fusion protein was purified into a buffer that containing either DPC or LMPG micelles (Figure 2B).

**Figure 3:**
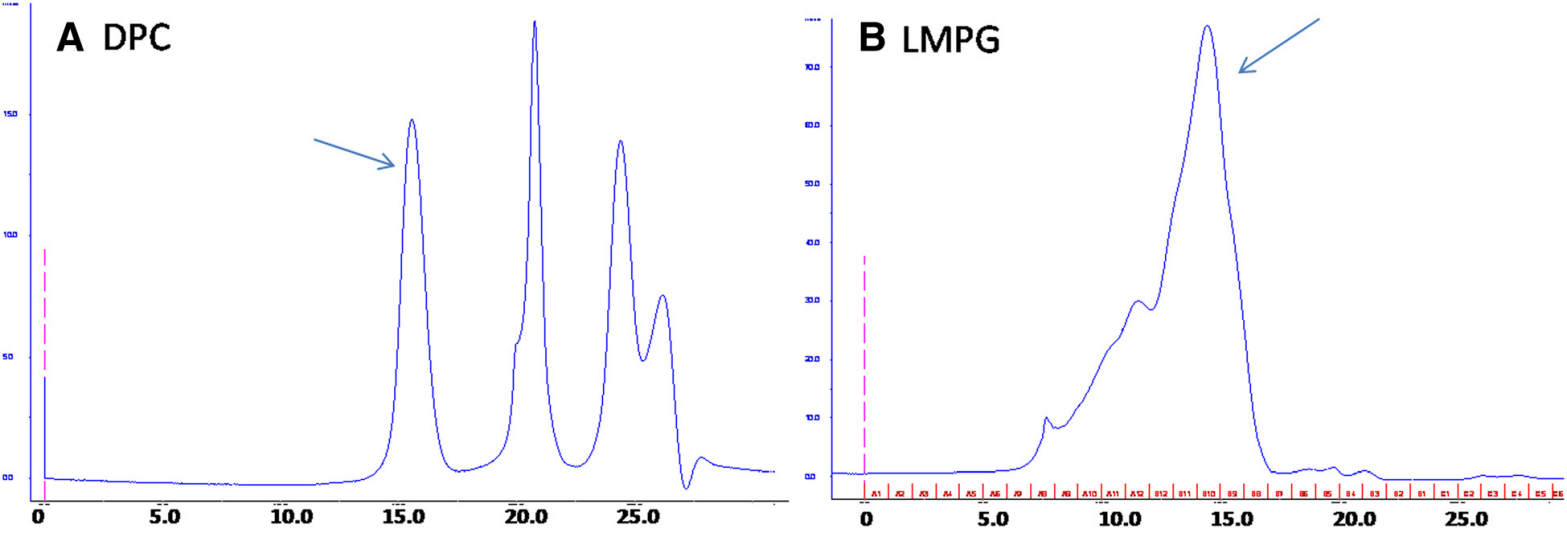
**Gel filtration chromatography of the purified protein.** (**A**) Gel filtration the p75^NTR^ TM domain DPC micelles. (**B**) Gel filtration of the p75^NTR^ TM domain in LMPG micelles. The peak indicated by arrow contains protein for further analysis.

To remove the KSI tag, the protein was buffer exchanged for digestion. Thrombin was added to a solution containing approximately 2 mg of protein. The digestion was verified by SDS-PAGE (Figure 2C). The uncleaved protein and the KSI tag was removed by passing the digestion mixture through a gravity column loaded with Ni^2+^-NTA resin that was pre-equilibrated with digestion buffer. Because a poly-histidine tag was present at the N-terminus of KSI, both the tag and the uncleaved protein should remain bound to the resin. The result showed that the p75^NTR^ TM domain could be purified using this procedure with more than 90% purity from SDS-PAGE analysis. The bands corresponding to the p75^NTR^ TM domain were verified by mass spectrometry (Figure 2B). To further purify and analyze oligomerization of the purified p75^NTR^ TM domain, the protein was loaded onto a gel filtration column. Homogenous protein was observed when p75^NTR^ TM domain was in the DPC micelles, whereas the protein seemed to exist in multiple conformations or aggregates when it was LMPG micelles (Figure 3). Using this method, we obtained about 1 mg of ^13^ C/^15^ N-labeled protein from 1 l of *E.coli* cell culture.

### CD analysis of the purified protein

**Figure 4:**
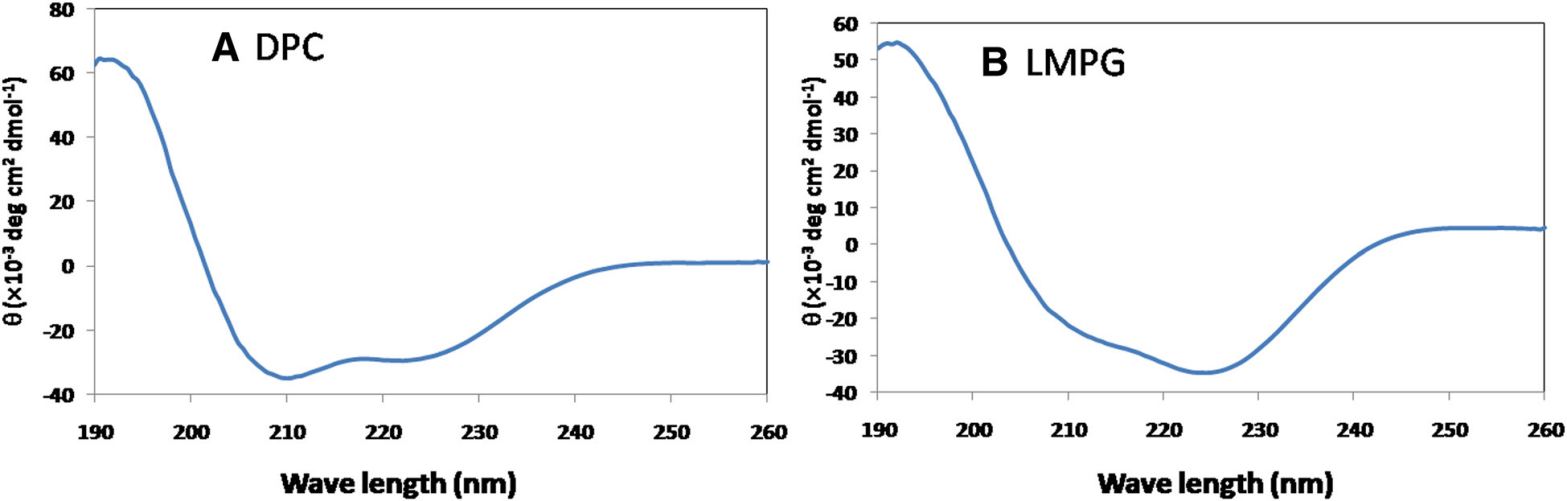
**CD spectra of the purified p75**^**NTR**^**TM domain in DPC and LMPG micelles.** The CD spectra were collected as described in the Materials and Methods. θ, molar residue ellipticity.

Although the purified protein was soluble in the detergent micelle, the folding of the target protein need to be further investigated. CD spectroscopy was used to analyze the secondary structure of the purified p75^NTR^ TM domain. Protein in both DPC and LMPG micelles was analyzed at 25°C using a CD spectrometer. Under these conditions, the CD spectrum of purified protein in DPC micelles exhibited a broad minimum at 208–228 nm and a positive peak at ~190 nm, which is consistent with helical secondary structure (Figure 4). Secondary analysis showed that the p75^NTR^ TM domain had ~70% alpha helical structure in DPC micelles. The CD spectrum of protein in LMPG micelles exhibited a different spectrum than that in DPC micelles indicating the different alpha helical composition in these two detergent micelles. The higher absorbance at 208 nm indicated the presence of less alpha helical component in LMPG micelles than in DPC micelles.

### NMR spectra of the purified protein

**Figure 5:**
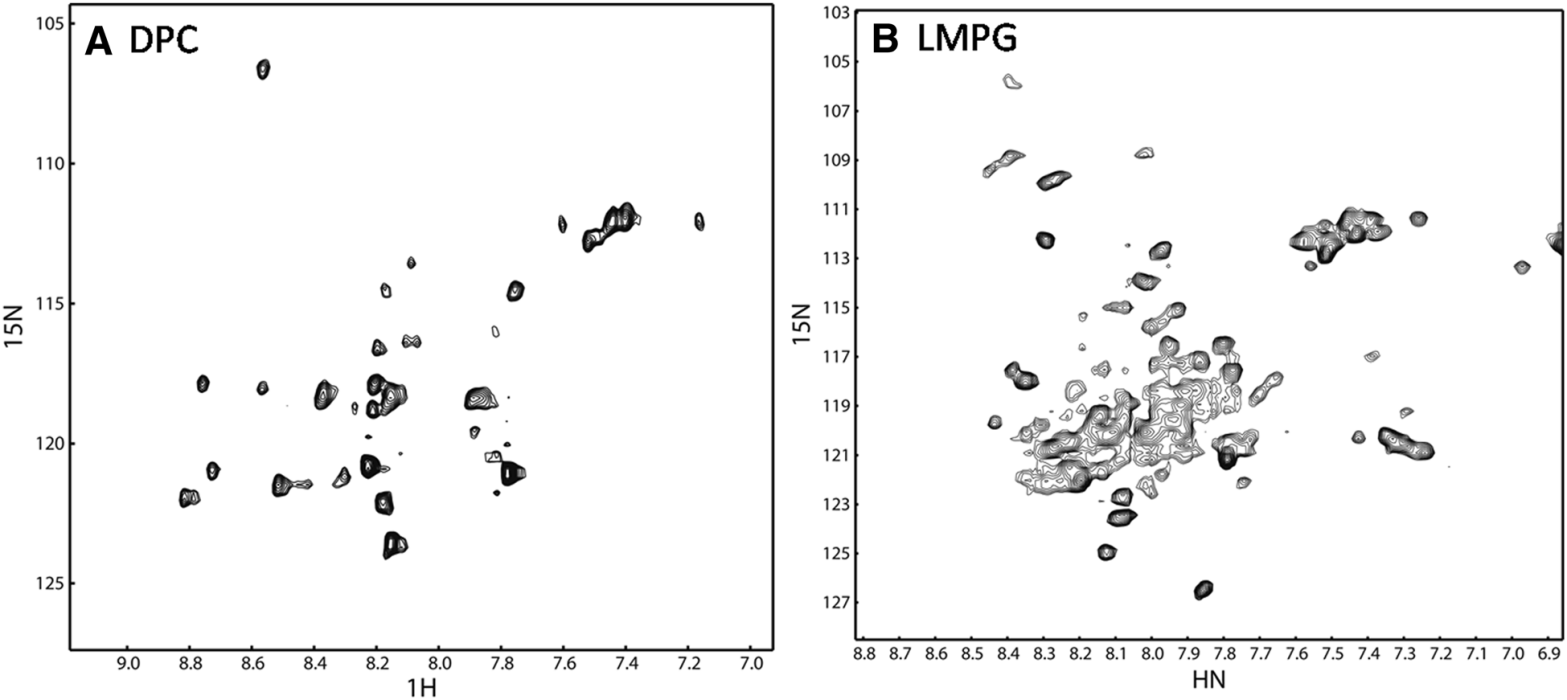
**HSQC spectra of the purified p75**^**NTR**^**TM domain.** (**A**) HSQC spectrum of the protein in DPC micelles. (**B**) HSQC spectrum of the protein in LMPG micelles. The protein concentration was 0.2 mM and the spectra were taken at 298 K on a Bruker 700 MHz magnet equipped with a cryoprobe.

Uniformly ^15^ N-labeled p75^NTR^ TM domain in DPC and LMPG micelles were analyzed to determine whether the purified protein could be analyzed using NMR spectroscopy. The quality and number of the cross peaks present in an HSQC spectrum provide information about whether the protein exists as a monomer or oligomer. The HSQC spectrum of the p75^NTR^ TM domain in DPC micelles showed nicely resolved cross peaks with the exception that there were fewer peaks than expected (Figure 5A). The LMPG micelles produced a spectrum with more cross peaks and many broaden peaks (Figure 5B). Peak broadening is normally caused by the oligomerization, which leads to a slower tumbling rate. The DPC detergent employed here provides nicely dispersed cross peaks in HSQC spectra for many membrane proteins. The p75^NTR^ TM domain exhibited less peaks in DPC micelles, which may be attributed to the fact that TM domain exist as a dimer that was observed using SDS-PAGE (Figure 2D).

## Discussion

Neurotrophins play important roles in nervous system development and function [21]. It was demonstrated that neurotrophins interact with dimer of the p75^NTR^ protein with unknown mechanism. Study showed that the Cys 257 in the TM domain of p75^NTR^ protein was important for the dimerization, which might be important for the neurotrophin-dependent receptor activity [19]. Structural study on the TM domain of the p75^NTR^ protein will provide insight into understanding the dimerization and its role in nervous system. As the TM domain of p75^NTR^ contains 20–30 residues, NMR spectroscopy will be a useful tool for its structural study. With purified samples in detergent micelles, further structural studies will be performed.

**Figure 6:**
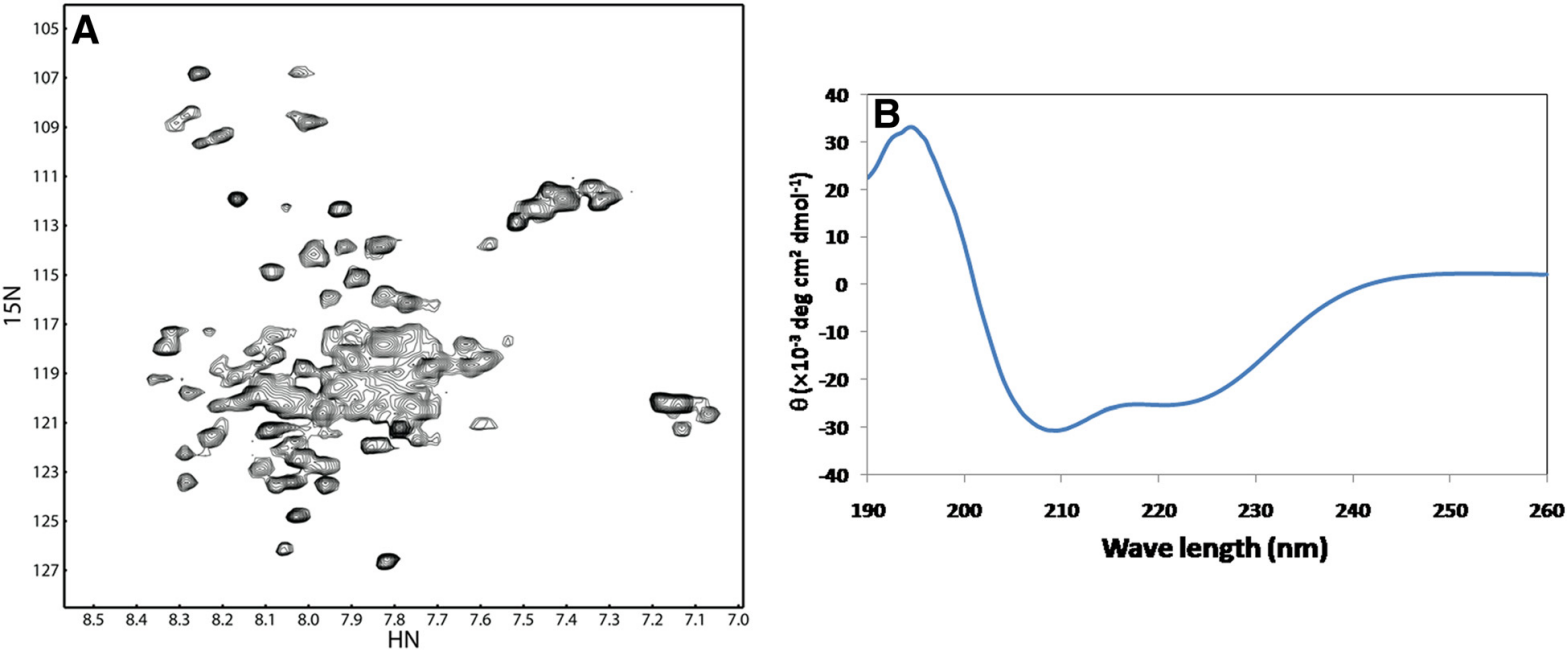
**NMR and CD spectra of purified protein in mixed micelles.** (**A**) HSQC spectrum of the purified p75^NTR^ TM domain in mixed micelles containing 2% LMPG and 2% SDS. The protein concentration was 0.2 mM and the spectrum was taken at 313 K on a Bruker 700 MHz magnet equipped with a cryoprobe. (**B**) CD spectrum of the purified p75^NTR^ TM domain in mixed micelles containing LMPG and SDS. The CD spectrum was collected as described in the Materials and Methods. θ, molar residue ellipticity.

Detergent micelles are very important for the extraction and purification of membrane proteins. In NMR study of membrane proteins, DPC and LMPG were frequently used [7]. In addition, mixed micelles containing different types of detergents were also useful in membrane protein structural studies [22,23]. In this study, we compared the NMR spectra of the purified p75^NTR^ TM domain in DPC and LMPG micelles. Further detergent screening is needed to improve the quality of the spectrum, for example, purified protein exhibited a spectrum with well dispersed cross peaks when it was in a mixed detergents containing both 2% LMPG and 2% SDS (Figure 6A). In addition, CD spectrum of protein in LMPG and SDS mixed micelles exhibited a broad minimum at 208–228 nm, which demonstrated higher composition of α-helical structures than that in LMPG micelles (Figure 6B).

There were two strategies for obtaining recombinant single TM domains of membrane proteins using affinity tags. One is expressing target protein with short tag and structural study was conducted directly for the tagged recombinant protein. This strategy requires that the fusion tag has no effect on the function and structure of the target protein. For example, the KCNE1 structure was determined using a construct containing a histidine tag at the N-terminus [14]. The other strategy is expressing target protein with fusion tags such as KSI [24]. The fusion tag was then removed by digestion of the recombinant protein with protease or chemicals [10,24]. KSI was used as a fusion tag for several TM domains of membrane proteins, in which chemical digestion was applied to remove the fusion tag for structural studies. As the size of a TM domain is small, the effect of fusion tag might affect its folding in micelles or other systems. Structures of several TM domains have been studied in the absence of fusion tag. In this study, KSI was also used as a fusion tag for the expression and purification of p75^NTR^ TM from *E.coli*. During recombinant protein purification, KSI fusion protein was refolded into detergent micelles and was removed after cleavage by thrombin that was confirmed to be active in several detergents that were commonly used in structural studies [16]. This method allowed us to obtain the p75^NTR^ TM domain for structural analysis using CD and NMR spectroscopy. Although the efficiency of KSI tag removal for thrombin was lower than that for CNBr, this method provided an environmental friendly way for protein production because special treatment and equipments will be necessary when CNBr was used in the biological lab. This method can be useful for production of protein with a single TM domain for NMR study.

## Conclusions

Our purification results demonstrated that the recombinant protein containing a KSI tag and the p75^NTR^ TM domain was expressed from inclusion bodies. The purified protein was refolded into micelles before thrombin cleavage (Figure 2B). Thrombin was active in the DPC and LMPG micelles, which made the recombinant protein cleavage possible without using CNBr or acid. The purified TM domain had both monomeric and dimeric forms by SDS-PAGE analysis (Figure 2D), which was consistent with the functional study [19]. The HSQC spectra were collected for the ^15^ N-labeled TM domain in detergent micelles. Although additional detergent screening need to be conducted to obtain a well-resolved HSQC spectrum for the p75^NTR^ TM domain for structural studies, our results indicated that this method may be universally applicable to the expression and purification of short peptides derived from the TM domain of a protein to avoid protein cleavage with CNBr or acid.

## Materials and methods

### Materials

The DNA polymerase and the restriction enzymes for molecular cloning were purchased from New England Biolabs (Beverly, USA). The pET-29b and pET-31b plasmids were purchased from Merck (Germany). *E.coli* BL21 (DE3) was purchased from Stratagene (La Jolla, USA). The fast protein liquid chromatography (FPLC) system, thrombin, S200 gel filtration column and PD10 desalting column were purchased from GE Healthcare (Uppsala Sweden). The SDS-PAGE system, the NuPAGE® gels, the running buffer, the competent cells and the SDS-PAGE molecular weight standard were obtained from Invitrogen (Carlsbad, USA). The Ni^2+^- nitrilotriacetic acid (NTA) affinity resin, the PCR purification kit and the plasmid extraction kit were purchased from Qiagen (Gmbh, Germany). Isopropyl β-D-1-thiogalactopyranoside (IPTG), dithiothreitol (DTT) and detergents were purchased from Anatrace (Maumee, USA) or Avanti Polar Lipids (Birmingham, USA). The ^15^NH_4_Cl, ^13^ C-glucose and D_2_O were obtained from Cambridge Isotope Laboratories (Andover, USA). All other chemicals used in this study were purchased from Sigma – Aldrich (St. Louis, USA).

### Molecular cloning

The plasmid for the expression of the KSI fusion protein was prepared as follows: cDNA coding for KSI with an N-terminal-decahistidine tag was synthesized with a C-terminal BamHI restriction site. The DNA encoding the TM domain of p75^NTR^ was synthesized with a BamHI and an XhoI restriction site at the N-terminus and C-terminus, respectively. The KSI DNA digested with NdeI and BamHI and the DNA encoding the TM domain of p75^NTR^ digested with BamHI and Xho I were ligated to pET-29b digested with Nde I and Xho I to generate a new plasmid: pE-T29-KSI- p75^NTR^. For the DNA encoding the p75^NTR^ TM domain, we chose to express approximately 50 amino acids containing the TM domain [19] and extra residues at its C- and N-termini. The insertion of the desired sequence into the plasmid was confirmed by DNA sequencing.

### Protein purification

The protein induction and purification was similar to previous work with single-span membrane proteins [25]. The plasmid was transformed into *E.coli* BL21 (DE3) competent cells and grown on LB plates containing 30 μg/ml of kanamycin. One to three colonies were picked and incubated in 50 ml of M9 medium. The overnight culture was transferred into 1 l of M9 medium with same antibiotic. When OD_600_ reached to 0.8, the protein was induced with 1 mM IPTG and left in the shaker for additional shaking at 200 rpm for 16 h at 37°C. The ^15^ N/^13^ C labeled protein was produced by growing *E.coli* cells in M9 with 1 g/l of ^15^ N NH_4_Cl and 2 g/l of ^13^ C-glucose. The *E. coli* were harvested by centrifugation at 8,000 ×g for 10 min at 4°C. The cell pellets were re-suspended into a lysis buffer at pH 7.8 that contained 20 mM Tris–HCl, 300 mM NaCl, and 2 mM β-mercaptoethanol and were then lysed by sonication on ice. The cell lysate was cleared by centrifugation at 40,000 ×g for 20 min. The resulting pellet was dissolved in a denaturing buffer at pH 7.8 that contained 20 mM Tris–HCl, 300 mM NaCl, 8 M urea, 0.2% SDS, 2 mM β-mercaptoethanol by rotating at room temperature for 3 h or overnight. The protein solution was then cleared by centrifugation at 40,000 × g for 20 min at room temperature. The supernatant was loaded onto 3 ml of Ni^2+^-NTA resin in a gravity column. Resin was first washed with 30 ml of denaturing buffer to remove nonspecifically bound proteins. The resin was then washed with 30 ml of SDS buffer at pH 7.8 that contained 20 mM Tris–HCl, 150 mM NaCl, 0.2% SDS and 2 mM β-mercaptoethanol to remove the urea. To exchange SDS with DPC or LMPG micelles, the resin was further washed with 30 ml of buffer that either 0.1% DPC or 0.1% LPMG in place of SDS in the SDS buffer. The protein was eluted with an elution buffer containing 300 mM imidazole, pH 6.5 and 0.1% DPC or 0.1%LMPG. The purified protein was used for the subsequent digestion experiments.

### Removal of the KSI tag

The purified protein buffer was exchanged with a digestion buffer containing 20 mM Tris–HCl, pH7.8, 150 mM NaCl, 2 mM CaCl_2_, 1 mM DTT, 0.1% DPC(LMPG) using a PD10 buffer exchange column. To digest 2 mg of protein, 20 units of thrombin protease was added to the protein solution and digestion was conducted overnight at room temperature. The digestion result was confirmed by SDS-PAGE. The protein solution was passed through a gravity column with 3 ml of Ni^2+^-NTA resin to remove the uncleaved protein and the KSI tag. The flow-through fraction containing the protein of interest was retained for further analysis.

### Gel filtration analysis

The fraction from the previous digestion step was concentrated and loaded onto a Superdex™ 200 10/300 GL gel filtration column. In this step, a gel filtration buffer was used, which was compatible with the future NMR study. This buffer contained 20 mM sodium phosphate, pH 6.5, and 0.1% DPC (or LMPG). The purification was conducted with a flow rate of 0.5 ml/min.

### Circular dichroism analysis

The circular dichroism (CD) experiments were performed on a Chirascan™ CD Spectrometer at 25°C. The CD spectrum of the p75^NTR^ TM domain at a concentration of 0.2 mg/ml was recorded in a buffer containing 20 mM sodium phosphate, pH 7.0, 0.5% of DPC (or LMPG), and 2 mM β-mercaptoethanol. The instrument was first blanked using a 0.1-cm path length quartz cuvette contained only buffer (without protein). The CD signal was acquired in the continuous mode with a 1-nm data pitch and a 1-nm bandwidth.

### NMR spectroscopy

The protein was exchanged to a buffer containing 20 mM sodium phosphate, pH 6.5, 1.0% detergent, 1 mM DTT and 10% D_2_O. The sample was concentrated to 200 μl of approximately 0.2 mM protein using an Amicon Ultra Centrifugal Filter with a 10,000 Da molecular weight cut off and put into a 3 mm NMR tube. The ^1^ H-^15^ N-HSQC spectra were collected using a Bruker 700 MHz magnet equipped with a cryoprobe at 313 K. The NMR spectra were processed with NMRPipe [26] and visualized with NMRView [27].

## Abbreviations

KSI = Ketosteroid isomerase; TM = Transmembrane; p75NTR = p75 Neurotrophin receptor; SDS-PAGE = Sodium dodecyl sulfate polyacrylamide gel electrophoresis; SDS = Sodium dodecyl sulfate; DPC = Dodecylphosphocholine; LMPG = Lyso-myristoyl phosphatidylglycerol.

## Competing interests

The authors declare that they have no competing interests.

## Authors’ contributions

CK designed the experiment. QL, AC and SG conducted and analyzed the data. CK drafted the manuscript. All the authors read and approved the final manuscript.
